# Effects of artemisinin on proliferation and apoptosis of human liver cancer HepG2 cells: A protocol of systematic review and meta-analysis

**DOI:** 10.1097/MD.0000000000020290

**Published:** 2020-05-15

**Authors:** Jing Li, Zhi-ye Liu, Hai-bo Yu, Qing Xue, Xiu-sheng Qu

**Affiliations:** a Department of Physiology, Jiamusi University School of Basic Medical Sciences; b Department of Chemotherapy and Radiotherapy, First Affiliated Hospital of Jiamusi University; c Department of Cardiology, First Affiliated Hospital of Jiamusi University; d Clinical Medicine of Class 7 in Grade 2016, Jiamusi University, Jiamusi, 154002, China.

**Keywords:** artemisinin, effect, human liver cancer HepG2 cells

## Abstract

**Background:**

This study will examine the effects of artemisinin on proliferation and apoptosis of human liver cancer HepG2 cells (HLCHG-2C).

**Methods:**

This study will systematically retrieve potential literatures in MEDLINE, Scopus, Web of Science, Cochrane Library, EMBASE, WANGFANG, and China National Knowledge Infrastructure from their initiation to the February 29, 2020. There are not limitations related to the language and publication time. All case-controlled studies (CCSs) or randomized controlled studies (RCSs) will be included in this study which investigated the effects of artemisinin on proliferation and apoptosis of HLCHG-2C. Two independent investigators will examine searched records, collect data from included studies, and will identify their methodological quality. Any divergences will be disentangled by discussion with another investigator. RevMan 5.3 software will be placed to pool the data and to carry out data analysis.

**Results:**

This study will summarize all eligible studies to test the effects of artemisinin on proliferation and apoptosis of HLCHG-2C.

**Conclusion:**

The results of this study will exert evidence to examine the effects of artemisinin on proliferation and apoptosis of HLCHG-2C, and it may benefit further research, patients, and healthcare providers.

**Systematic review registration:**

INPLASY202040075.

## 1 Introduction

Primary liver cancer (LC) is one of the most common malignant tumors.^[1,2,3,4]^ Its incidence is about 1 million new cases each year globally.^[5,6]^ Of that, hepatocellular carcinoma (HCC) accounts for about 90% of all LC.^[7]^ Thus, it is very important to treat patients with HCC. A variety of treatments have reported to treat patients with HCC, their efficacy is still not limited.^[8,9,10,11,12,13,14]^ Thus, it is very urgent to find more effective medications to treat such condition.

Previous study reported that cellular immunotherapy has been increasingly used for the treatment of HCC.^[15]^ Recent experimental studies have found that artemisinin can manage the proliferation and apoptosis of human liver cancer HepG2 cells (HLCHG-2C),^[16,17,18,19,20,21,22,23]^ which is a potential candidate for HCC therapy in the clinical practice. However, no systematic review is identified to appraise the effects of artemisinin on proliferation and apoptosis of HLCHG-2C.

## 2 Methods

### 2.1 Study registration

This study was registered and funded on INPLASY202040075, and it has been reported based on the guidelines of the Preferred Reporting Items for Systematic Reviews and Meta-Analysis (PRISRMA) Protocol statement.^[24,25]^

### 2.2 Eligibility criteria

#### 2.2.1 Types of trials

All potential case-controlled studies (CCSs) or randomized controlled studies (RCSs) will be included, which explore the effects of artemisinin on proliferation and apoptosis of HLCHG-2C. We will not consider any other studies, such as review, comment, and uncontrolled studies.

#### 2.2.2 Types of subjects

This study will include HLCHG-2C as its targeted subject.

#### 2.2.3 Types of exposures/interventions

In the experimental group, all HLCHG-2C were managed using artemisinin. However, we will exclude studies that utilized combination of artemisinin with other treatments.

In the control group, all treatment options were available for HLCHG-2C. However, we will not consider studies employed any types of artemisinin as their control managements.

#### 2.2.4 Types of outcome measurements

Primary outcome is proliferation and apoptosis of HLCHG-2C. Its proliferation is examined by cell viability test, and its apoptosis is detected by flow cytometry.

Secondary outcomes include HLCHG-2C proliferation and apoptosis related-proteins and genes expression. The proteins (including β-catenin, PTEN, Akt, CyclinD1, P27, Bax, Bcl-2, Caspase-3, Caspase-9, Cytochrame-C, and PCNA) are measured by immunofluorescence or western blot test. The genes (including Mcl-1 and Caspase-3) are identified by Real-time polymerase chain reaction or relevant test.

### 2.3 Literature sources and search strategy

Electronic databases will be systematically searched potential studies in MEDLINE, Scopus, Web of Science, Cochrane Library, EMBASE, WANGFANG, and China National Knowledge Infrastructure from their initiation to the February 29, 2020, regardless language and publication time restrictions. We will include all potential CCSs or RCSs that identified the effects of artemisinin on proliferation and apoptosis of HLCHG-2C. A search strategy sample for MEDLINE is summarized (Table 1). We will also provide similar search strategies for other electronic databases.

**Table 1:**
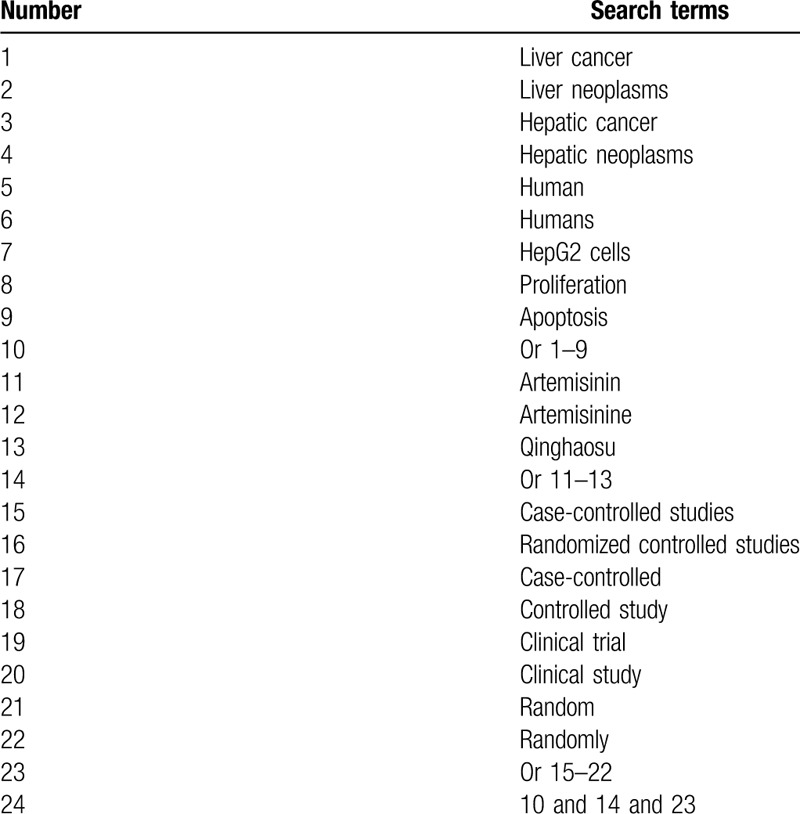
Search strategy of MEDLINE.

In addition, we will examine other sources to avoid missing more potential studies, such as Google Scholar, reports on related agencies, conference proceedings, and reference lists of relevant reviews.

### 2.4 Study selection

Two investigators will independently carry out primary article scanning according to the predefined eligibility criteria. First, titles/abstracts of sought literatures will be screened to eliminate unconnected studies and duplicates. Second, full papers of potential studies will be further examined in accordance with the full inclusion criteria. Any differences will be figured out by discussion with another investigator. The results of study selection will be shown in a PRISRMA flow diagram with details.

### 2.5 Data collection and management

Before data collection, a standardized data extraction sheet will be developed to collect relate study data from all eligible studies. Two investigators will independently collect following data: publication information (along with author name and publication time), HLCHG-2C information, study setting and design, interventions and comparators (such as names and types of managements, dosage, etc), outcomes, results, findings, and conflict of interest. Any disagreements will be worked out by consultation with another investigator. Any insufficient or missing information will be obtained from original authors by email or telephone.

### 2.6 Study quality assessment

The study quality will be independently appraised by 2 investigators using Newcastle-Ottawa Scale for CCSs and Cochrane risk of bias tool for RCSs. Any different opinions will be arbitrated with another investigator.

### 2.7 Statistical analysis

#### 2.7.1 Data synthesis

This study will apply RevMan 5.3 software to synthesize and analyze extracted data. We will calculate treatment effects of pooled dichotomous data as risk ratio and 95% confidence intervals (CIs), and synthesized continuous data as weighted mean difference or standardized mean difference and 95% CIs. Heterogeneity across studies will be assessed using Higgins *I*^2^ statistic. *I*^2^ ≤ 50% is considered as having minimal heterogeneity and data will be pooled using a fixed-effects model, while *I*^2^ > 50% is regarded as suggesting substantial heterogeneity and data will be synthesized using a random-effects model. When there is minimal heterogeneity, we will arrange to conduct a meta-analysis if possible. Otherwise, when there is significant heterogeneity, we will carry out a subgroup analysis to investigate possible sources of substantial heterogeneity.

#### 2.7.2 Subgroup analysis

If necessary, this study will carry out a subgroup analysis to test the sources of heterogeneity in accordance with the different types of studies, study quality, and intervention and controls.

#### 2.7.3 Sensitivity analysis

Whenever possible, this study will conduct a sensitivity analysis to examine the robustness and stability of study findings by removing low quality studies.

#### 2.7.4 Reporting bias

This study will plan to explore a funnel plot and Egger's regression test to identify reporting bias if over 10 studies are included.

### 2.8 Dissemination and ethics

This study will not analyze individual patient data, thus, no ethical approval will be provided. It will be disseminated on a peer-reviewed journal or conference presentation.

## 3 Discussion

Although previous studies have reported the effects of artemisinin on proliferation and apoptosis of HLCHG-2C,^[16,17,18,19,20,21,22,23]^ no study has accumulated sufficient evidence to draw definitive conclusions on this topic. Thus, this study will systematically and comprehensively assess the effects of artemisinin on proliferation and apoptosis of HLCHG-2C. The results of this study will provide helpful evidence to explore a potential candidate for HCC therapy in the clinical practice.

## Author contributions

**Conceptualization:** Jing Li, Zhi-ye Liu, Qing Xue.

**Data curation:** Hai-bo Yu, Xiu-sheng Qu.

**Formal analysis:** Jing Li, Zhi-ye Liu, Qing Xue.

**Investigation:** Xiu-sheng Qu.

**Methodology:** Hai-bo Yu, Qing Xue.

**Project administration:** Xiu-sheng Qu.

**Resources:** Jing Li, Zhi-ye Liu, Hai-bo Yu, Qing Xue.

**Software:** Jing Li, Zhi-ye Liu, Hai-bo Yu, Qing Xue.

**Supervision:** Xiu-sheng Qu.

**Validation:** Jing Li, Hai-bo Yu, Qing Xue, Xiu-sheng Qu.

**Visualization:** Zhi-ye Liu, Qing Xue, Xiu-sheng Qu.

**Writing – original draft:** Jing Li, Hai-bo Yu, Qing Xue, Xiu-sheng Qu.

**Writing – review & editing:** Jing Li, Zhi-ye Liu, Qing Xue, Xiu-sheng Qu.
